# Supplementary material for: Complexome profiling of the Chlamydomonas psb28 mutant reveals TEF5 as an early PSII assembly factor
Source: Plant Cell. 2025 Mar 17;37(6):koaf055. doi: 10.1093/plcell/koaf055 (PMC12164590; doi:10.1093/plcell/koaf055)
Supplement: koaf055_Supplementary_Data [file koaf055_supplementary_data.zip › psb28_interactive.zip!/plots/intensity/qualityPlots/peptides/MSD2_Cre13.g605150.t1.2.html]

### Cre13.g605150.t1.2

Trivial name: MSD2  
SeqCoverage [median]: 19.70  
Mass: 24.85 kDa  
  
 

| Sequence | MissedCleavage | Mass [Da] | wtIntensity | muIntensity | Score |
| --- | --- | --- | --- | --- | --- |
| ALEQQAEAEHKGDVAK | 1 | 1722.85 | 6425.03 | 5731.02 | 68.17 |
| ALIEAQWK | 0 | 957.53 | 0.00 | 500.34 | 62.84 |
| HHATYVANLNK | 0 | 1266.65 | 1559.33 | 2712.26 | 60.10 |
| LISLQSAIK | 0 | 971.60 | 3360.77 | 9547.60 | 107.69 |
|  |  |  | 11345.1 | 18491.2 |  |
